# T-helper Cell-Mediated Proliferation and Cytokine Responses against Recombinant Merkel Cell Polyomavirus-Like Particles

**DOI:** 10.1371/journal.pone.0025751

**Published:** 2011-10-03

**Authors:** Arun Kumar, Tingting Chen, Sari Pakkanen, Anu Kantele, Maria Söderlund-Venermo, Klaus Hedman, Rauli Franssila

**Affiliations:** 1 Departments of Virology, Haartman Institute, University of Helsinki, Helsinki, Finland; 2 Department of Bacteriology and Immunology, Haartman Institute, University of Helsinki, Helsinki, Finland; 3 Division of Infectious Diseases, Helsinki University Central Hospital, Helsinki, Finland; 4 Helsinki University Central Hospital Laboratory Division, Helsinki, Finland; The University of Hong Kong, Hong Kong

## Abstract

The newly discovered Merkel Cell Polyomavirus (MCPyV) resides in approximately 80% of Merkel cell carcinomas (MCC). Causal role of MCPyV for this rare and aggressive skin cancer is suggested by monoclonal integration and truncation of large T (LT) viral antigen in MCC cells. The mutated MCPyV has recently been found in highly purified leukemic cells from patients with chronic lymphocytic leukemia (CLL), suggesting a pathogenic role also in CLL. About 50–80% of adults display MCPyV-specific antibodies. The humoral immunity does not protect against the development of MCC, as neutralizing MCPyV antibodies occur in higher levels among MCC patients than healthy controls. Impaired T-cell immunity has been linked with aggressive MCC behavior. Therefore, cellular immunity appears to be important in MCPyV infection surveillance. In order to elucidate the role of MCPyV-specific Th-cell immunity, peripheral blood mononuclear cells (PBMC) of healthy adults were stimulated with MCPyV VP1 virus-like particles (VLPs), using human bocavirus (HBoV) VLPs and *Candida albicans* antigen as positive controls. Proliferation, IFN-γ, IL-13 and IL-10 responses were examined in 15 MCPyV-seropositive and 15 seronegative volunteers. With the MCPyV antigen, significantly stronger Th-cell responses were found in MCPyV-seropositive than MCPyV-seronegative subjects, whereas with the control antigens, the responses were statistically similar. The most readily detectable cytokine was IFN-γ. The MCPyV antigen tended to induce stronger IFN-γ responses than HBoV VLP antigen. Taken together, MCPyV-specific Th-cells elicit vigorous IFN-γ responses. IFN-γ being a cytokine with major antiviral and tumor suppressing functions, Th-cells are suggested to be important mediators of MCPyV-specific immune surveillance.

## Introduction

Merkel cell polyomavirus (MCPyV) discovered by Feng et al in 2008, is responsible for a rare, yet aggressive neuroendrocrine neoplasia, Merkel cell carcinoma (MCC) [1], [2], [3]. The virus has been shown to be present in 24–89% of MCCs in populations of varied geographic origins [4]–[7]. It has been shown to be integrated clonally into the MCC genome [1], [8]. Antibodies recognizing MCPyV tumor associated antigens appear to be a relatively specific MCC marker [9]. Recently, an association of MCPyV infection with chronic lymphocytic leukemia (CLL) was reported [10]–[12], yet the causal association remains to be proven. Serological studies have shown that 50–80% of adults display MCPyV-specific antibodies [13]–[15]. Very recently, the presence of MCPyV DNA sequences was reported in buffy coats of healthy blood donors pointing to latency/persistence in peripheral blood leukocytes [16], [17]. As MCPyV VLPs can elicit antibody responses, they have been suggested to be potential vaccine candidates [18]. However, as neutralizing MCPyV antibodies occour in high titers among patients, they apparently fail to prevent MCC tumorigenesis [18]. It is therefore possible that cell mediated immunity (CMI) may be involved in protection against MCPyV- induced malignancy. Our aim was to elucidate the strength and polarization of MCPyV-specific T-helper cell immunity among asymptomatic adults. T-helper cell mediated proliferation, interferon-gamma (IFN-γ), interleukin-10 (IL-10) and interleukin-13 (IL-13) responses were studied.

IFN-γ is a major antiviral cytokine, produced not only by Th1 cells but also by cytotoxic T-cells and NK cells [19]. It is a critical extrinsic tumor-suppressor factor in immunocompetent hosts and it has several types of antitumor actions [20]–[24]. IL-10 is an important anti-inflammatory cytokine [25] and its major sources are T-helper type 2 (Th2) cells and a subset of regulatory T-cells [26]. IL-10 inhibits Th1 cells, NK cells and macrophages. These three cell types are required for optimal pathogen clearance, and they also contribute to tissue damage during infection. In consequence, IL-10 can both impede pathogen clearance and ameliorate immunopathology [25]. The role of this cytokine on the immune response against cancer is controversial. As it can inhibit several key phenomena of adaptive immune responses, it has been considered to allow malignant cells to escape from immune surveillance [27], [28]. By contrast, there is data to suggest that IL-10 might also favour immune-mediated cancer rejection [29]–[32]. IL-13 is an important cytokine produced mainly by Th2 cells [33], [34]. It possesses several unique effector functions including regulation of gastrointestinal parasite expulsion, intracellular parasitism, airway hyperresponsiveness, allergic inflammation [35] and class switch to IgE and IgG4 [36]. The role of IL-13 in regulating tumor growth depends on the tumor cell type. In some models inhibition of IL-13 or IL-13 receptors has promoted tumor growth [37], [38] whereas in others tumor growth has been inhibited [38], [39]. In chronic B lymphocytic leukemia (B-CLL) models IL-13 has been shown to block apoptosis of tumor cells [40], [41].

In this study we demonstrate that vigorous MCPyV-specific Th cell responses are readily detectable in constitutionally healthy adults.

## Results

### Proliferation responses among MCPyV-seropositive and seronegative subjects

We determined MCPyV-specific T-cell proliferation in 15 MCPyV-seropositive and 15 seronegative subjects. Virus-specific proliferation responses of the MCPyV-seropositive subjects were much stronger than those of the seronegative subjects, both at 0.25 µg/ml and 2.5 µg/ml concentrations of antigen (Tables 1 and 2 and Fig. S1).

**Table 1:** Comparison of MCPyV-specific proliferation and cytokine responses among 15 MCPyV seropositive and −15 seronegative subjects at 0.25 µg/ml antigen concentration.

MCPyV serostatus	ΔCPM^a^±SD	IFN-γ pg/mL±SD	IL-10 pg/mL±SD	IL-13 pg/mL±SD
Positive	5878±5959	159.8±234.0	23.3±30.5	23.5±28.2
Negative	534±660	6.5±8.40	4.50±4.80	4.0±5.40
*P*	<0.0001	0.006	0.026	0.021

a Δ CPM: antigen-specific CPM- background.

**Table 2:** Comparison of MCPyV-specific proliferation and cytokine responses among MCPyV seropositive and –seronegative subjects at 2.5 µg/ml antigen concentration.

MCPyV serostatus	ΔCPM^a^	IFN-γ pg/mL	IL-10 pg/mL	IL-13 pg/mL
Positive	9562±7419	280.7±197.6	51.2±56.0	56.5±54.9
Negative	3086±3918	51.8±60.6	22.7±21.8	20.5±26.4
*P*	0.001	<0.0001	0.041	0.019

a Δ CPM: antigen-specific CPM- background.

The proliferation responses became more vigorous when the MCPyV-antigen concentration was elevated from 0.25 µg/ml to 2.5 µg/ml. The increase in mean response was ∼2 fold among the seropositive subjects, and even higher, ∼5 fold among the seronegative controls (Tables 1 and 2). These increases were statistically significant both among the seropositive (P = 0.011) and seronegative subjects (P = 0.002).

Six seropositive and none of the seronegative subjects were responders (having ΔCPM>5000) with the lower 0.25 µg/ml antigen concentration (P = 0.017). With the higher 2.5 µg/ml concentration, the corresponding numbers were 12 and three among the seropositive and seronegative subjects, respectively (P = 0.001) (data not shown). With the control antigens HBoV (Table 3) and *Candida albicans* (Table 4) no statistically significant differences in proliferation were found. With the responder criteria the control-antigen specific responses were statistically identical among the MCPyV-seropositive and seronegative subjects, P = 1.0 with both antigens (data not shown).

**Table 3:** Comparison of HBoV-specific proliferation and cytokine responses among MCPyV seropositive and –seronegative subjects at 2.5 µg/ml antigen concentration.

MCPyV serostatus	ΔCPM^a^	IFN-γ pg/mL	IL-10 pg/mL	IL-13 pg/mL
Positive	6704±7937	121.3±154.4	15.9±41.8	74.4±118.1
Negative	6884±11106	127.8±214.0	10.9±21.5	45.1±71.2
*P*	0.174	0.345	0.624	0.305

a Δ CPM: antigen-specific CPM- background.

**Table 4:** Comparison of *Candida albicans*-specific proliferation and cytokine responses among MCPyV seropositive and –seronegative subjects at 2.5 µg/ml antigen concentration.

MCPyV serostatus	ΔCPM^a^	IFN-γ pg/mL	IL-10 pg/mL	IL-13 pg/mL
Positive	42728±32744	1179.8±1367.8	481.3±387.9	225.2±186.0
Negative	30379±37614	1174.2±1318.1	661.4±456,3	183.7±230.6
*P*	0.106	0.486	0.285	0.305

a Δ CPM: antigen-specific CPM- background.

### IFN-γ, IL-10 and IL-13 responses among MCPyV-seropositive and seronegative subjects

MCPyV-specific IFN-γ, IL-10 and IL-13 responses were readily detectable even with the lower 0.25 µg/ml antigen concentration among the seropositive subjects, and they were significantly stronger than the corresponding responses among the seronegative subjects (Table 1).

With the 2.5 µg/ml MCPyV antigen concentration the average cytokine responses were higher than with the 0.25 µg/ml concentration (Tables 1 and 2), and the differences were statistically significant with all the cytokines, both among the seropositive (P≤0.011) and seronegative (P≤0.018) subjects.

Also with the higher MCPyV antigen concentration the cytokine responses were stronger among the seropositive subjects than among the seronegative ones. The difference was particularly evident with IFN-γ, and also significant with IL-10 and IL-13 (Table 2 and Fig. S1).

With the control antigens the cytokine responses were very similar among the MCPyV seropositive and seronegative subjects. The P-values with the HBoV and *Candida albicans* antigens were ≥0.305 and ≥0.285, respectively (Tables 3 and 4).

As seen in Tables 1 and 2, IFN-γ was the dominant MCPyV-associated cytokine. Also at individual level, a response pattern of IFN-γ>IL-10 and IFN-γ>IL-13 was often detected with both antigen concentrations (P≤0.014). Among the seronegative subjects this pattern was not borne at MCPyV VLP concentration of 0.25 µg/ml (P≥0.444), whereas at the higher concentration also the seronegative subjects tended to show higher responses with IFN-γ than with IL-10 (P = 0.069) or with IL-13 (P = 0.047).

With the positive control antigen HBoV (Table 3) IFN-γ responses were also higher than the corresponding IL-10 or IL-13 responses, both among the seropositive (P≤0.030) and seronegative subjects (P≤0.026).

Finally, we compared the same MCPyV-seropositive subjects' MCPyV-derived IFN-γ responses with the same subjects' HBoV-derived IFN-γ responses. Significantly stronger IFN-γ responses were detected with MCPyV-antigen than with HBoV antigen (P = 0.016) (data not shown).

### Identification of the proliferating and cytokine secreting cells

**Figure 1:**
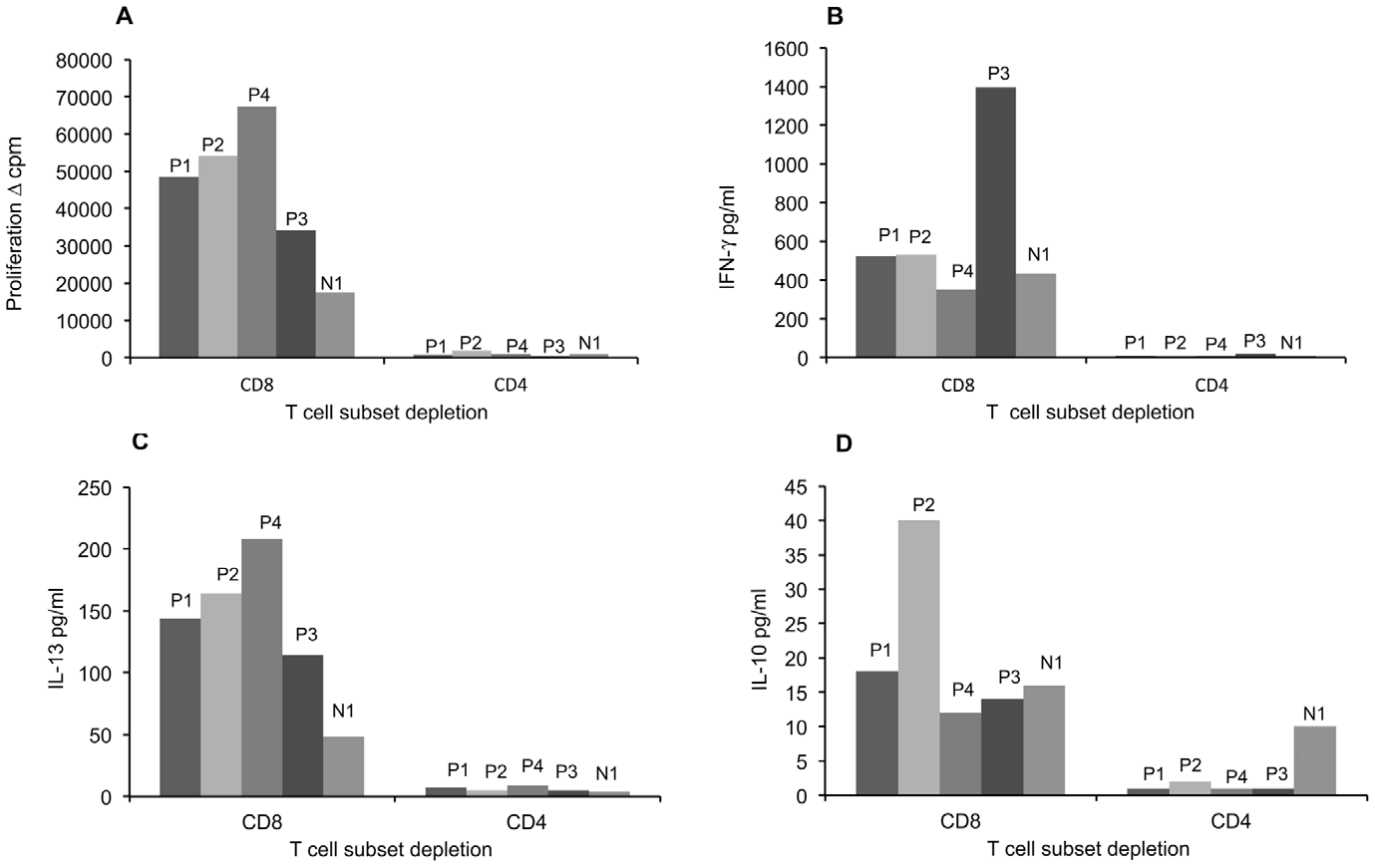
MCPyV VP1–specific T cell responses of MCPyV seropositive individuals after T cell subset depletion. PBMC of four MCPyV seropositive (P1 to P4) and one seronegative subject with strong MCPyV-specific CMI (N1) were depleted of either CD4+ or CD8+ T cells and stimulated with MCPyV VP1-VLPs (2.5 µg/ml). Proliferation (panel A) and cytokine (IFN-γ, IL-13 and IL-10) responses (panel B, C, D) were studied by thymidine incorporation and ELISA, respectively.

To identify the proliferating and cytokine secreting cell populations, the PBMC were depleted either of CD4+ or CD8+ T cells by using monoclonal antibodies (MAbs) attached to magnetic beads. Seropositive subjects P1 to P4 and a seronegative subject N1 who had constantly shown strong responses with MCPyV were studied. MCPyV-specific proliferation, IFN-γ, IL-10 and IL-13 secretion was readily detectable after depletion of CD8+ T cells, whereas the removal of CD4+ T cells strongly reduced the responses among all the subjects (Fig. 1).

### HLA restriction of cytokines and proliferating cells

**Figure 2:**
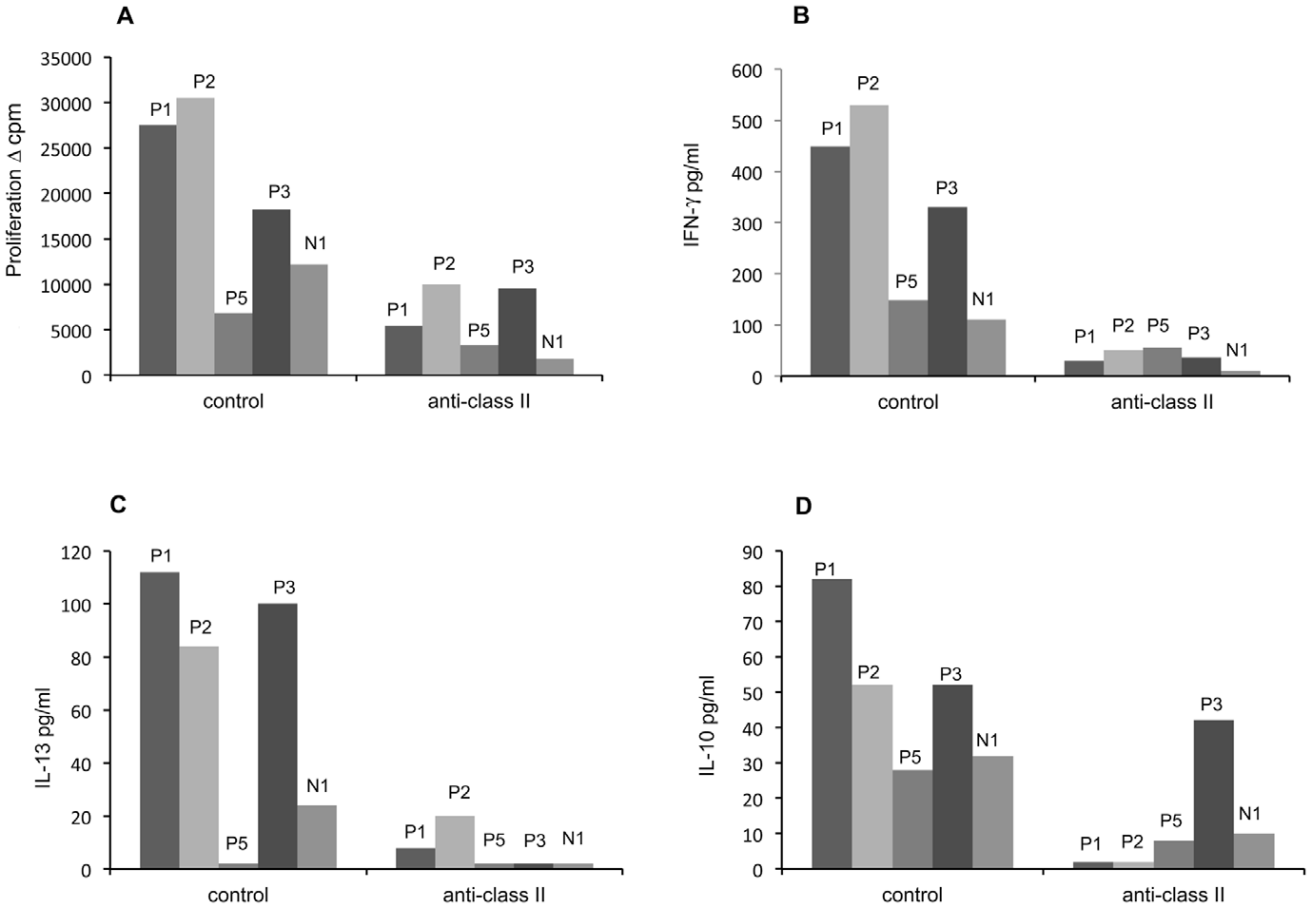
Effect of HLA class II-specific monoclonal antibodies (MAbs) on MCPyV-VP1 specific proliferation and cytokine responses. PBMC from four MCPyV seropositive subjects (P1 to P3 and P5) and from a seronegative subject (N1) were incubated either with a HLA class II-specific blocking MAb or with an isotype-matched control MAb. The effect of these MAbs on MCPyV-specific (2.5 µg/ml) proliferation (panel A) and cytokine (panels B, C and D) responses are shown. Subjects P1 to P3 and N1 are same than in Figure 1.

HLA class restriction of the cytokine and proliferation responses were studied with a class II-specific MAb (which blocks antigen presentation) and with an isotype-matched control MAb. Seropositive subjects (P1, P2, P3 and P5) together with a seronegative subject (N1) were studied. With the isotype control MAb proliferation and cytokine responses were readily detectable, whereas they were invariably reduced with the HLA class II-specific MAb (Fig. 2).

## Discussion

**Figure 3:**
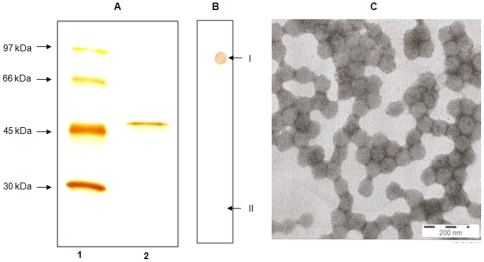
Characterization of MCPyV VP1 antigens. Silver staining of capsid protein (panel A) in 10% SDS PAGE. Lane 1: molecular weight markers, lane 2: MCPyV VP1 capsid antigen. Dot blotting (panel B) for MCPyV antigen, studied with MCPyV-IgG positive (I) and negative (II) sera. Electron microscopy of sterile MCPyV particles (panel C) purified by caesium chloride density gradient ultracentrifugation, with 200 nm scale bar shown.

A significant proportion of human population has encountered MCPyV. Because neutralizing MCPyV antibodies occur in high titers in patients with MCC [18], it is likely that infection surveillance is not completely mediated by humoral immunity. Instead, cell mediated immune mechanisms may play a central role, yet they have not been explored so far. The present study is the first to report on cell-mediated immunity against MCPyV. The responses were regarded to be highly specific for MCPyV, as the studies were carried out by using highly purified (Fig. 3) VLPs. T-cell subset depletion and HLA class II blocking showed that the main sources of MCPyV-specific proliferation and cytokine responses were CD4+ Th-cells, not the cells of innate immunity, even in a MCPyV seronegative subject (N1) showing strong cellular responses to MCPyV.

The concentration of the MCPyV-antigen used in the assay had a significant importance. With the lower concentration a “classical” response pattern was observed: Th-cell proliferation and cytokine responses were largely confined within the seropositive subjects. However, with the higher concentration, MCPyV-specific responses were detectable also among some seronegative subjects. This type of responders have been previously denoted as “immune seronegative” subjects in a herpes simplex virus model [42], [43]. With both antigen concentrations the seropositive subjects nevertheless had significantly stronger MCPyV-specific Th-cell responses than the seronegative subjects had, whereas Th-cell responses against the control antigens HBoV and *Candida albicans* were statistically similar in the two groups. The presence of MCPyV- seronegative responders suggests that B-cell immunity against MCPyV is not always persistent, or that a degree of cross-reactivity in the VP1 Th-cell epitopes may exist between MCPyV and some hitherto-unidentified virus. VP1 proteins of other polyomaviruses are possible candidates. For instance, the VP1 protein of a recently discovered trichodysplasia spinulosa-associated polyomavirus (TSPyV) has as high as 50.6% amino-acid similarity with that of MCPyV [44]. Alternatively, some MCPyV strains might be of aberrant B-cell antigenicity. One such MCPyV strain, termed “350”, having critical double mutations at VP1 positions 288 and 316, has been described to date [45]. VP1 of strain “350” is not recognized by sera strongly reactive with VP1s of MCPyV strains lacking these mutations [45].

Furthermore, it remains possible that the MCPyV-reactive Th cells have been originally primed by pathogens possessing largely different T cell epitopes. This is because a very high level of crossreactivity is an essential feature of the T-cell receptor [46]–[49].

We found that MCPyV specific Th-cells secrete the Th2-like cytokine IL-13, the regulatory-like cytokine IL-10 and the Th1-like cytokine IFN-γ. MCPyV-specific IL-10 may have importance in regulation of humoral immunity (Note S1). IFN-γ was the most readily detectable cytokine with MCPyV, and the responses were significantly stronger than the corresponding responses with the HBoV positive-control antigen, highlighting the strength of this response. This is the main finding of our study, due to the tumor-suppressing and antiviral functions of this cytokine. Of note, cellular infiltration and cytokine mRNA (including IFN-γ) have been studied in MCC biopsies. Arany and Tyring found lacking of IFN-γ responses in MCC biopsies [50], whereas Kelly et al found an association between favorable prognosis and the presence of elevated expression of IFN-γ mRNA [51]. It should be noted that in these studies the antigen specificity of tumor infiltrating lymphocytes was not determined.

Taken together, the antigen-specific Th-cell responses of healthy individuals in the present study along with studies showing a lack of Th-cell responses on a general level in patients with MCC suggest a central role for CMI in infection surveillance of MCPyV. The imperative next step is to study MCPyV-related diseases such as MCC or CLL for antigen-specific CMI to get further evidence of the pathogenic importance of MCPyV-specific Th immunity.

## Materials and Methods

### Study groups

Altogether randomly selected 30 asymptomatic subjects (age range 25–58 years) were studied: 15 were seropositive and 15 seronegative for MCPyV. This study protocol followed the human experimentation guidelines of the US Department of Health and Human Services in the conduct of clinical research and was approved by the ethics committee of the Department of Medicine in Helsinki University Central Hospital. Written informed consent was obtained from all volunteers.

### Antigens for proliferation and cytokine assays

MCPyV VP1 and HBoV VP2 capsids were expressed with recombinant baculoviruses in Sf9 cells and purified by CsCl gradient ultracentrifugation [52]–[54]. After extensive dialysis the protein was concentrated and purified further by using 50 KDa MWCO centrifugal filters (Amicon Ultra, Millipore, Billerica, MA). The antigens were further characterized by silver staining (SilverXpress, Invitrogen, Carlsbad, CA, USA) (Fig. 3 A) and dot blotting (Fig. 3 B) with MCVPy seropositive human sera as described earlier [52], [53]. The purity for MCPyV protein was >90% by densitometry (Gel Doc 2000 Gel Documentation systems with Quantity One Quantitation Software, Bio-Rad). Electron microscopy with negative staining showed virus-like particles (Fig. 3 C). As a second control antigen, we used in-house prepared and heat inactivated *Candida albicans*. Endotoxin in the antigen preparations was measured by the Limulus amebocyte lysate assay (QCL-1000; Cambrex Biosciences, Walkersville, MD, USA), and it was less than 2 EU/mg with MCPyV and HBoV antigens.

### Antibody assay

We measured MCPyV and HBoV IgG in plasma by EIA, employing as antigen virus-like particles [52], [54].

### Isolation of PBMC

Blood was drawn to mononuclear cell separation tubes (Vacutainer CPT, Becton Dickinson, Franklin Lakes, NJ, USA). The tubes were centrifuged at 1500× g for 30 minutes and washed two times with PBS. PBMC were separated within 2 hrs of blood sampling followed by counting [55].

### Lymphocyte culture

For lymphocyte culture, isolated PBMC were resuspended in RPMI-1640 (Sigma, St Louis, MO, USA) containing 20 mM HEPES, 2 mM L-glutamine, streptomycin (100 µg/ml), penicillin (100 U/ml), 50 µM 2-mercaptoethanol and 10% human AB serum (Cambrex Biosciences, USA) and were cultured with the antigens [55]. MCPyV VLP were used at 0.25 µg/ml and 2.5 µg/ml and the HBoV VLP and *Candida albicans* control antigens at 2.5 µg/ml.

### Proliferation assay

Counted PBMC and antigens in triplicate were placed in 96 well U-bottom plates (Coster, Corning Inc., Corning, NY, USA). Cells (200,000/well) were cultured for 6 days (37°C; 5% CO_2_) and pulsed for the last 16 hours with 1 µCi of tritiated thymidine (specific activity 50 Ci/mmol; Nycomed Amersham, Buckinghamshire, UK). Thymidine incorporation was measured in a liquid scintillation counter (Microbeta, Wallac,Turku, Finland). The data were expressed as counts per minute (Δ cpm): Δ cpm = mean cpm (test antigen)−mean cpm (media) [53], [55].

### Cytokine assays

PBMC culture supernatants were harvested after 3 days for IFN-γ and after 5 days for IL-10 and IL-13, and were stored at −20°C. Cytokine production in the supernatants was analysed by IFN-γ, IL-10 (Pharmingen, San Diego, CA, USA) and IL-13 (Invitrogen corporation CA, USA) kits, according to the manufacturers' instructions. Background (media) cytokine production was subtracted from total to yield antigen specific cytokine production. The detection limits for IFN-γ, IL-10 and IL-13 were 5, 8 and 6 pg/ml, respectively.

### Depletion of CD4+ or CD8+ cells

PBMC were depleted of CD4+ or CD8+ T cells by using magnetic beads coated with CD4- or CD8-specific monoclonal antibodies (Invitrogen Dynal AS, Oslo, Norway), according to the manufacturer's instructions. Then, 200,000 pure CD4+ or CD8+ depleted cells were cultured with the antigens as described [55].

### Antibody blocking assays

Class restriction of the T-cell responses was further studied by HLA class II-specific MAbs (HLA-DR, DP, DQ) (IgG2a, clone Tu39; BD PharMingen), or isotype control MAb (IgG2a, clone G155- 178; BD PharMingen). These antibodies were used at 5 µg/ml, according to the manufacturer's instructions.

### Statistical methods

Responses among MCPyV seropositive and seronegative subjects were compared by using the Mann-Whitney U test. Paired responses were evaluated by using the Wilcoxon Signed Rank test. The distribution of responders having Δ cpm>5000 [42], [43] against each antigen was studied using Fisher's Exact test. P values<0.05 were considered significant. All analyses were done with a SPSS statistical program version 15.0.

## Supporting Information

Figure S1 **Cytokine and proliferation responses in the 15 MCPyV seropositve (A) and 15 seronegative (B) subjects with the 2.5 µg/ml MCPyV antigen (▪) and media (□).**
(PDF)

Figure S2 **Cytokine responses versus µg/ml MCPyV IgG titers in the 15 seropositive subjects.** Responses from a seropositive subject with strong MCPyV-specific cytokine responses but low titers of MCPyV IgG are shown with an open triangle (Δ).
(PDF)

Note S1 **Antibody versus cytokine responses in 15 MCPyV seropositive subjects.**
(PDF)

## References

[1] Feng H, Shuda M, Chang Y, Moore PS (2008). Clonal integration of a polyomavirus in human Merkel cell carcinoma..

[2] Kassem A, Schopflin A, Diaz C, Weyers W, Stickeler E (2008). Frequent detection of Merkel cell polyomavirus in human Merkel cell carcinomas and identification of a unique deletion in the VP1 gene.. Cancer Res.

[3] Andres C, Belloni B, Puchta U, Sander CA, Flaig MJ (2010). Prevalence of MCPyV in Merkel cell carcinoma and non-MCC tumors.. J Cutan Pathol.

[4] Foulongne V, Kluger N, Dereure O, Brieu N, Guillot B (2008). Merkel cell polyomavirus and Merkel cell carcinoma, france.. Emerg Infect Dis.

[5] Becker JC, Houben R, Ugurel S, Trefzer U, Pfohler C (2009). MC polyomavirus is frequently present in Merkel cell carcinoma of european patients.. J Invest Dermatol.

[6] Garneski KM, Warcola AH, Feng Q, Kiviat NB, Leonard JH (2009). Merkel cell polyomavirus is more frequently present in north american than australian Merkel cell carcinoma tumors.. J Invest Dermatol.

[7] Paolini F, Donati P, Amantea A, Bucher S, Migliano E (2011). Merkel cell polyomavirus in Merkel cell carcinoma of italian patients.. Virol J.

[8] Shuda M, Feng H, Kwun HJ, Rosen ST, Gjoerup O (2008). T antigen mutations are a human tumor-specific signature for Merkel cell polyomavirus.. Proc Natl Acad Sci U S A.

[9] Paulson KG, Carter JJ, Johnson LG, Cahill KW, Iyer JG (2010). Antibodies to Merkel cell polyomavirus T antigen oncoproteins reflect tumor burden in merkel cell carcinoma patients.. Cancer Res.

[10] Koljonen V, Kukko H, Pukkala E, Sankila R, Bohling T (2009). Chronic lymphocytic leukaemia patients have a high risk of Merkel-cell polyomavirus DNA-positive Merkel-cell carcinoma.. Br J Cancer.

[11] Pantulu ND, Pallasch CP, Kurz AK, Kassem A, Frenzel L (2010). Detection of a novel truncating Merkel cell polyomavirus large T antigen deletion in chronic lymphocytic leukemia cells.. Blood.

[12] Teman CJ, Tripp SR, Perkins SL, Duncavage EJ (2011). Merkel cell polyomavirus (MCPyV) in chronic lymphocytic leukemia/small lymphocytic lymphoma.. Leuk Res.

[13] Kean JM, Rao S, Wang M, Garcea RL (2009). Seroepidemiology of human polyomaviruses.. PLoS Pathog.

[14] Tolstov YL, Pastrana DV, Feng H, Becker JC, Jenkins FJ (2009). Human Merkel cell polyomavirus infection II. MCV is a common human infection that can be detected by conformational capsid epitope immunoassays.. Int J Cancer.

[15] Touze A, Le Bidre E, Laude H, Fleury MJ, Cazal R (2011). High levels of antibodies against Merkel cell polyomavirus identify a subset of patients with Merkel cell carcinoma with better clinical outcome.. J Clin Oncol.

[16] Pancaldi C, Corazzari V, Maniero S, Mazzoni E, Comar M (2011). Merkel cell polyomavirus DNA sequences in the buffy coats of healthy blood donors.. Blood.

[17] Mertz KD, Junt T, Schmid M, Pfaltz M, Kempf W (2010). Inflammatory monocytes are a reservoir for Merkel cell polyomavirus.. J Invest Dermatol.

[18] Pastrana DV, Tolstov YL, Becker JC, Moore PS, Chang Y (2009). Quantitation of human seroresponsiveness to Merkel cell polyomavirus.. PLoS Pathog.

[19] Boehm U, Klamp T, Groot M, Howard JC (1997). Cellular responses to interferon-gamma.. Annu Rev Immunol.

[20] Kaplan DH, Shankaran V, Dighe AS, Stockert E, Aguet M (1998). Demonstration of an interferon gamma-dependent tumor surveillance system in immunocompetent mice.. Proc Natl Acad Sci U S A.

[21] Muller-Hermelink N, Braumuller H, Pichler B, Wieder T, Mailhammer R (2008). TNFR1 signaling and IFN-gamma signaling determine whether T cells induce tumor dormancy or promote multistage carcinogenesis.. Cancer Cell.

[22] Kornacker M, Moldenhauer G, Herbst M, Weilguni E, Tita-Nwa F (2006). Cytokine-induced killer cells against autologous CLL: Direct cytotoxic effects and induction of immune accessory molecules by interferon-gamma.. Int J Cancer.

[23] Tannenbaum CS, Hamilton TA (2000). Immune-inflammatory mechanisms in IFNgamma-mediated anti-tumor activity.. Semin Cancer Biol.

[24] Beatty GL, Paterson Y (2001). Regulation of tumor growth by IFN-gamma in cancer immunotherapy.. Immunol Res.

[25] Couper KN, Blount DG, Riley EM (2008). IL-10: The master regulator of immunity to infection.. J Immunol.

[26] Mosser DM, Zhang X (2008). Interleukin-10: New perspectives on an old cytokine.. Immunol Rev.

[27] Mocellin S, Marincola FM, Young HA (2005). Interleukin-10 and the immune response against cancer: A counterpoint.. J Leukoc Biol.

[28] Mapara MY, Sykes M (2004). Tolerance and cancer: Mechanisms of tumor evasion and strategies for breaking tolerance.. J Clin Oncol.

[29] Zheng LM, Ojcius DM, Garaud F, Roth C, Maxwell E (1996). Interleukin-10 inhibits tumor metastasis through an NK cell-dependent mechanism.. J Exp Med.

[30] Kundu N, Beaty TL, Jackson MJ, Fulton AM (1996). Antimetastatic and antitumor activities of interleukin 10 in a murine model of breast cancer.. J Natl Cancer Inst.

[31] Kaufman HL, Rao JB, Irvine KR, Bronte V, Rosenberg SA (1999). Interleukin-10 enhances the therapeutic effectiveness of a recombinant poxvirus-based vaccine in an experimental murine tumor model.. J Immunother.

[32] Berman RM, Suzuki T, Tahara H, Robbins PD, Narula SK (1996). Systemic administration of cellular IL-10 induces an effective, specific, and long-lived immune response against established tumors in mice.. J Immunol.

[33] Cherwinski HM, Schumacher JH, Brown KD, Mosmann TR (1987). Two types of mouse helper T cell clone. III. further differences in lymphokine synthesis between Th1 and Th2 clones revealed by RNA hybridization, functionally monospecific bioassays, and monoclonal antibodies.. J Exp Med.

[34] McKenzie AN, Culpepper JA, de Waal Malefyt R, Briere F, Punnonen J (1993). Interleukin 13, a T-cell-derived cytokine that regulates human monocyte and B-cell function.. Proc Natl Acad Sci U S A.

[35] Wynn TA (2003). IL-13 effector functions.. Annu Rev Immunol.

[36] Punnonen J, Aversa G, Cocks BG, McKenzie AN, Menon S (1993). Interleukin 13 induces interleukin 4-independent IgG4 and IgE synthesis and CD23 expression by human B cells.. Proc Natl Acad Sci U S A.

[37] Kapp U, Yeh WC, Patterson B, Elia AJ, Kagi D (1999). Interleukin 13 is secreted by and stimulates the growth of hodgkin and reed-sternberg cells.. J Exp Med.

[38] Terabe M, Park JM, Berzofsky JA (2004). Role of IL-13 in regulation of anti-tumor immunity and tumor growth.. Cancer Immunol Immunother.

[39] Ma HL, Whitters MJ, Jacobson BA, Donaldson DD, Collins M (2004). Tumor cells secreting IL-13 but not IL-13Ralpha2 fusion protein have reduced tumorigenicity in vivo.. Int Immunol.

[40] Chaouchi N, Wallon C, Goujard C, Tertian G, Rudent A (1996). Interleukin-13 inhibits interleukin-2-induced proliferation and protects chronic lymphocytic leukemia B cells from in vitro apoptosis.. Blood.

[41] Zaninoni A, Imperiali FG, Pasquini C, Zanella A, Barcellini W (2003). Cytokine modulation of nuclear factor-kappaB activity in B-chronic lymphocytic leukemia.. Exp Hematol.

[42] Posavad CM, Wald A, Hosken N, Huang ML, Koelle DM (2003). T cell immunity to herpes simplex viruses in seronegative subjects: Silent infection or acquired immunity?. J Immunol.

[43] Posavad CM, Remington M, Mueller DE, Zhao L, Magaret AS (2010). Detailed characterization of T cell responses to herpes simplex virus-2 in immune seronegative persons.. J Immunol.

[44] van der Meijden E, Janssens RW, Lauber C, Bouwes Bavinck JN, Gorbalenya AE (2010). Discovery of a new human polyomavirus associated with trichodysplasia spinulosa in an immunocompromized patient.. PLoS Pathog.

[45] Carter JJ, Paulson KG, Wipf GC, Miranda D, Madeleine MM (2009). Association of Merkel cell polyomavirus-specific antibodies with Merkel cell carcinoma.. J Natl Cancer Inst.

[46] Wucherpfennig KW, Strominger JL (1995). Molecular mimicry in T cell-mediated autoimmunity: Viral peptides activate human T cell clones specific for myelin basic protein.. Cell.

[47] Hemmer B, Vergelli M, Gran B, Ling N, Conlon P (1998). Predictable TCR antigen recognition based on peptide scans leads to the identification of agonist ligands with no sequence homology.. J Immunol.

[48] Lang HL, Jacobsen H, Ikemizu S, Andersson C, Harlos K (2002). A functional and structural basis for TCR cross-reactivity in multiple sclerosis.. Nat Immunol.

[49] Mycko MP, Waldner H, Anderson DE, Bourcier KD, Wucherpfennig KW (2004). Cross-reactive TCR responses to self antigens presented by different MHC class II molecules.. J Immunol.

[50] Arany I, Tyring SK (1998). Status of cytokine and antigen presentation genes in Merkel cell carcinoma of the skin.. J Cutan Med Surg.

[51] Paulson KG, Iyer JG, Tegeder AR, Thibodeau R, Schelter J (2011). Transcriptome-wide studies of Merkel cell carcinoma and validation of intratumoral CD8+ lymphocyte invasion as an independent predictor of survival.. J Clin Oncol.

[52] Chen T, Hedman L, Mattila PS, Jartti T, Ruuskanen O (2011). Serological evidence of Merkel cell polyomavirus primary infections in childhood.. J Clin Virol.

[53] Kumar A, Filippone C, Lahtinen A, Hedman L, Soderlund-Venermo M (2011). Comparison of th-cell immunity against human bocavirus and parvovirus B19: Proliferation and cytokine responses are similar in magnitude but more closely interrelated with human bocavirus.. Scand J Immunol.

[54] Soderlund-Venermo M, Lahtinen A, Jartti T, Hedman L, Kemppainen K (2009). Clinical assessment and improved diagnosis of bocavirus-induced wheezing in children, finland.. Emerg Infect Dis.

[55] Franssila R, Hedman K (2004). T-helper cell-mediated interferon-gamma, interleukin-10 and proliferation responses to a candidate recombinant vaccine for human parvovirus B19.. Vaccine.

