# Supplementary figures and images for: T-helper Cell-Mediated Proliferation and Cytokine Responses against Recombinant Merkel Cell Polyomavirus-Like Particles
Source: PLoS One. 2011 Oct 3;6(10):e25751. doi: 10.1371/journal.pone.0025751 (PMC3185038; doi:10.1371/journal.pone.0025751)

Fig. S1

A

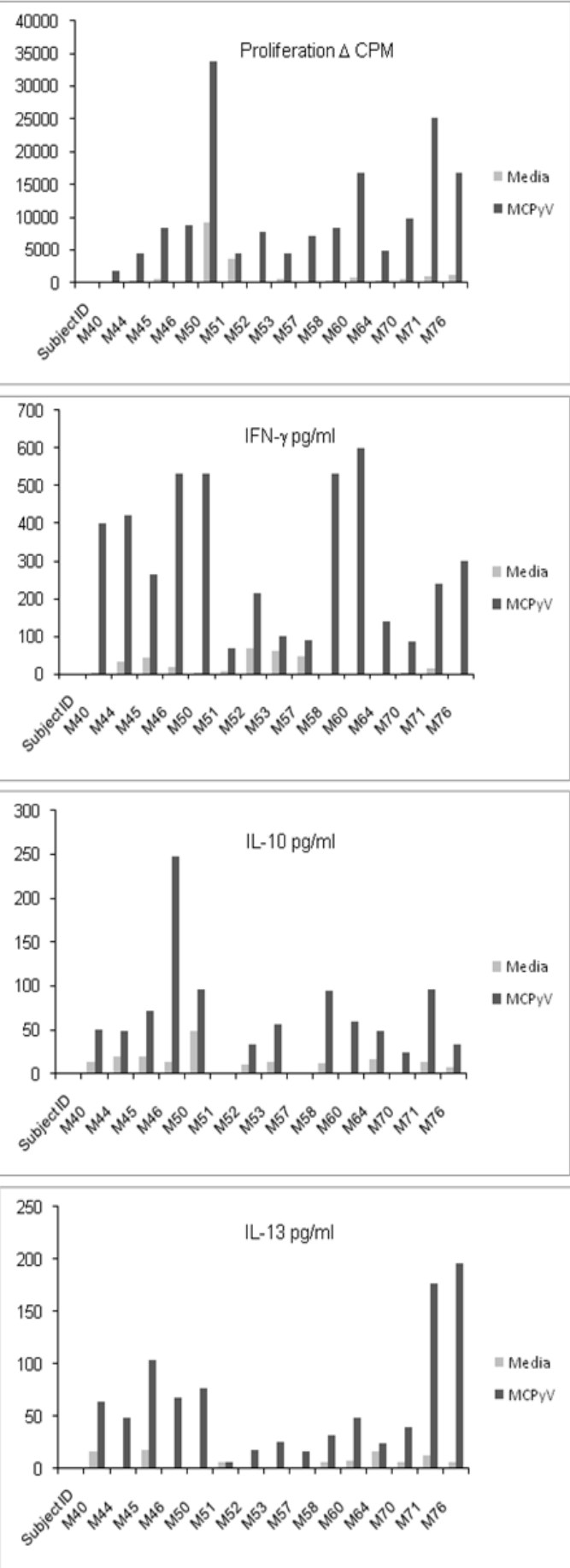

B

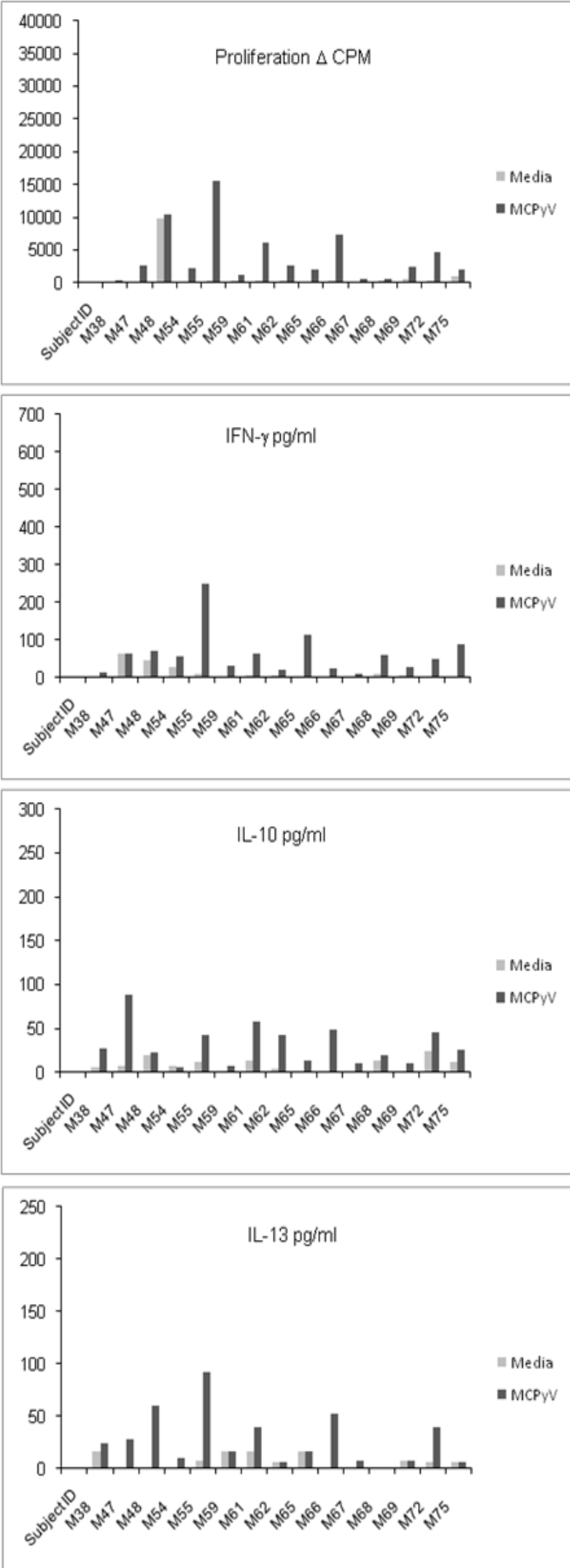

Supplement: Figure S1 — Cytokine and proliferation responses in the 15 MCPyV seropositve (A) and 15 seronegative (B) subjects with the 2.5 µg/ml MCPyV antigen (▪) and media (□). (PDF) [file pone.0025751.s001.pdf]

Fig. S2

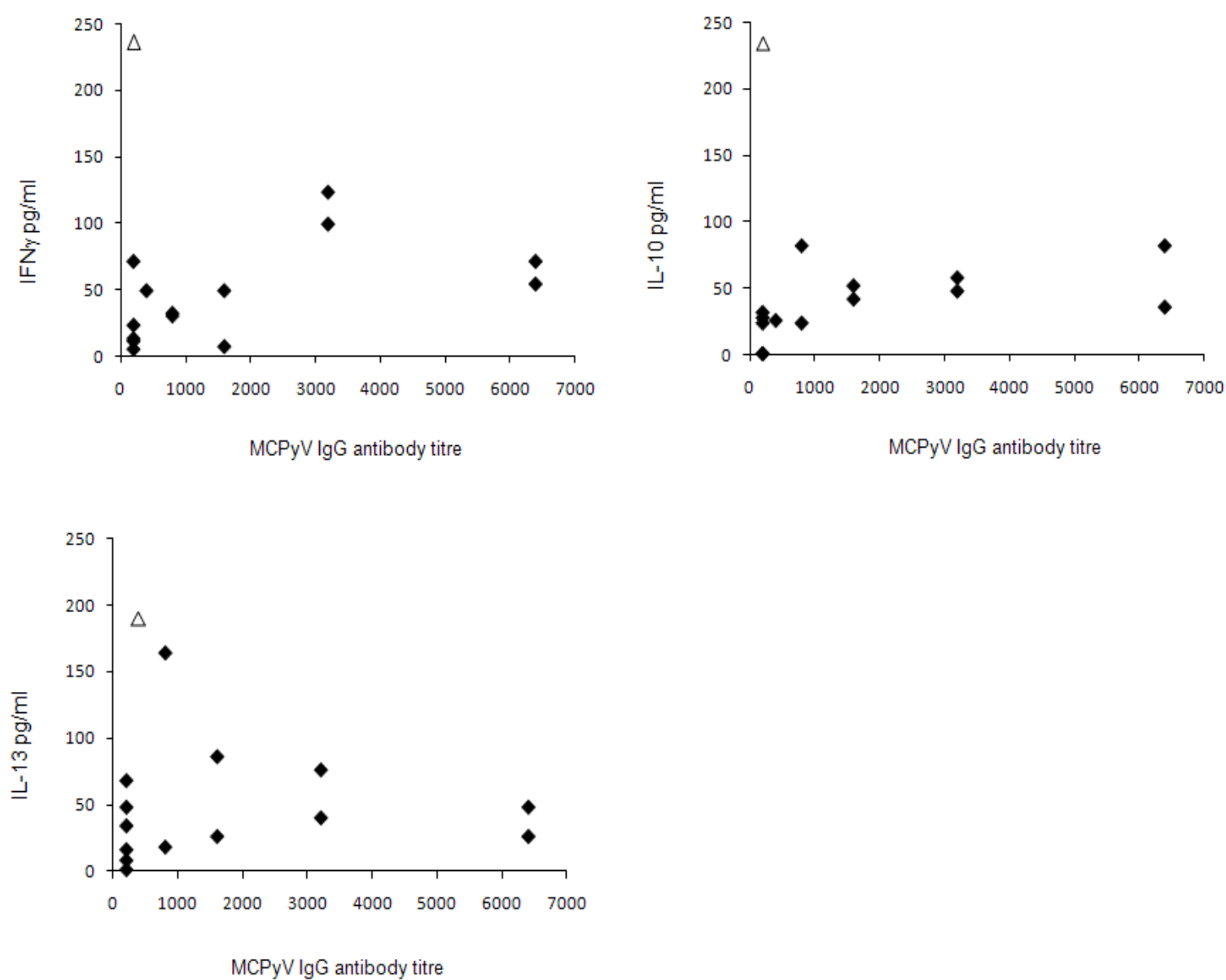

Supplement: Figure S2 — Cytokine responses versus µg/ml MCPyV IgG titers in the 15 seropositive subjects. Responses from a seropositive subject with strong MCPyV-specific cytokine responses but low titers of MCPyV IgG are shown with an open triangle (Δ). (PDF) [file pone.0025751.s002.pdf]
