# Supplementary material for: T-helper Cell-Mediated Proliferation and Cytokine Responses against Recombinant Merkel Cell Polyomavirus-Like Particles
Source: PLoS One. 2011 Oct 3;6(10):e25751. doi: 10.1371/journal.pone.0025751 (PMC3185038; doi:10.1371/journal.pone.0025751)
Supplement: Note S1 — Antibody versus cytokine responses in 15 MCPyV seropositive subjects. (PDF) [file pone.0025751.s003.pdf]

Note S1. Antibody versus cytokine responses in 15 MCPyV seropositive subjects

To study the effect of CMI to the level of MCPyV-IgG, we plotted each seropositive subjects MCPyV-IgG titers versus MCPyV-specific cytokine responses. MCPyV-antigen was used at 2.5 µg/ml. Surprisingly, the highest MCPyV-specific IFN-γ, IL-10 and IL-13 responses were found in an individual with only low levels of MCPyV-IgG, and consequently, no significant correlation between IgG titer and IFN-γ, IL-10 or IL-13 were detected ($P \geq 0.620$). However, if this "topresponder" was omitted from analysis, a positive correlation between MCPyV-specific IgG and IL-10 ($P = 0.046$) was detected in the remaining 14 MCPyV seropositive subjects, whereas the correlations with IFN-γ ($P = 0.053$) and IL-13 ($P = 0.757$) still remained nonsignificant (Fig. S2). Correlation was studied with Spearman´s test.
